# Noise exposure levels predict blood levels of the inner ear protein prestin

**DOI:** 10.1038/s41598-022-05131-z

**Published:** 2022-01-21

**Authors:** Ashley Parker, Kourosh Parham, Erika Skoe

**Affiliations:** 1 Department of Speech, Language, and Hearing Sciences, University of Connecticut, Storrs, CT USA; 2 Division of Otolaryngology-Head and Neck Surgery, Department of Surgery, University of Connecticut Health, Farmington, CT USA; 3 Connecticut Institute for Brain and Cognitive Sciences, University of Connecticut, Storrs, CT USA

**Keywords:** Physiology, Medical research

## Abstract

Serological biomarkers of inner ear proteins are a promising new approach for studying human hearing. Here, we focus on the serological measurement of prestin, a protein integral to a human’s highly sensitive hearing, expressed in cochlear outer hair cells (OHCs). Building from recent nonhuman studies that associated noise-induced OHC trauma with reduced serum prestin levels, and studies suggesting subclinical hearing damage in humans regularly engaging in noisy activities, we investigated the relation between serum prestin levels and environmental noise levels in young adults with normal clinical audiograms. We measured prestin protein levels from circulating blood and collected noise level data multiple times over the course of the experiment using body-worn sound recorders. Results indicate that serum prestin levels have a negative relation with noise exposure: individuals with higher routine noise exposure levels tended to have lower prestin levels. Moreover, when grouping participants based on their risk for a clinically-significant noise-induced hearing loss, we found that prestin levels differed significantly between groups, even though behavioral hearing thresholds were similar. We discuss possible interpretations for our findings including whether lower serum levels may reflect subclinical levels of OHC damage, or possibly an adaptive, protective mechanism in which prestin expression is downregulated in response to loud environments.

## Introduction

Over 460 million individuals across the globe suffer from debilitating hearing impairment^1^, yet the disability is vastly underassessed in primary care exams^2,3^. Serological biomarkers, involving the sampling and analysis of blood, are a promising new approach to addressing this unmet hearing health need, with the potential to help detect noise-induced hearing loss (NIHL) and other forms of acquired hearing loss earlier than ever before through the analysis of inner ear proteins in circulation. Direct access to the inner ear is not feasible in live humans without anesthetic preparation. However, a blood-based biopsy done in the upper limb through standard venipuncture approaches could potentially improve acquired hearing loss detection because it utilizes one of the most common tools in clinical medicine—the blood draw—giving new insight into intricacies and dynamics of the human ear and hearing. Candidate biomarkers must go through intensive vetting and validation before clinical trials or diagnostic use.

**Figure 1:**
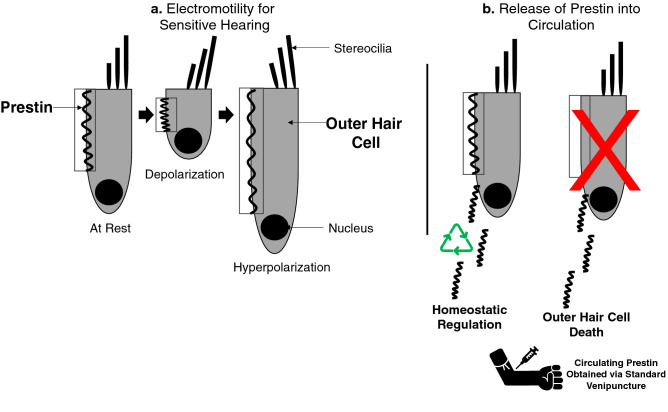
Highly simplified schematic depiction of outer hair cell (OHC) somatic electromotility and mechanisms for circulating prestin. (**a**) Prestin generates somatic electromotility, the contraction and elongation of OHCs in response to changes in transmembrane potential. This electromotility underlies acute auditory sensitivity. (**b**) Two postulated triggers for prestin entering the bloodstream: prestin is continuously recycled as part of the homeostatic regulation of cochlear function (left), and prestin enters circulation after outer hair cell death (right).

Understanding how to interpret blood-based biomarkers in the healthy, undamaged ear is a requisite for using such techniques to gain insight into the biological dynamics of the damaged ear. The human ear is an intricate biological system, complete with its own built-in hearing-aid that increases mechanical vibrations in the inner ear to make low-intensity sounds louder. This amplifier is vital to everyday communication, and without it, the acoustic richness of speech and music would likely be imperceptible. The “cochlear amplifier” is powered by a unique motor protein, prestin, that is highly expressed in the cochlear outer hair cells (OHCs)^4^ (see Ref.^4^ for a review on prestin, and Ref.^5^ for a review on the cochlear amplifier). As part of the cochlear amplification of sounds, OHCs undergo rapid changes in length (Fig. 1), created by conformational changes to prestin molecules that reside in the lateral membrane of OHC^8^, generating a force that adds energy into the cochlear partition. This somatic electromotility is crucial for sensitive hearing^6,7^. Therefore, it is not surprising that OHC loss is a significant cause of age-related hearing deficiency and NIHL (see Ref.^9^).

For prestin^10^ to be measured in blood serum from the upper limb, prestin molecules must pass the cochlear labyrinth and enter the circulatory system. The timeline and mechanisms are complex and still under investigation (see Ref.^11^ for a discussion on the possible turnover timeline). The release of prestin into circulation might be trigged by two conditions that are not necessarily mutually exclusive (Fig. 1). The first is that cellular components of OHCs are released into the blood stream after the phagocytosis of damaged OHCs^12–14^. The second is that it happens as part of homeostatic regulation. That is, prestin may be removed from the inner ear as part of the normal proteomic recycling (turnover) processes that maintain cellular homeostasis. Consistent with this proposed homeostatic mechanism, we have previously shown that the healthy ear has stable levels of prestin molecules in circulation^15^. Possible mechanisms for uptake, recycling, and sorting of plasma-membrane proteins, such as prestin in the OHCs. (For a review, see Ref.^16^).

We have previously used animal models to demonstrate changes to serum prestin after a traumatic noise event that resulted in hearing loss from OHC death^17,18^. A parallel literature is also emerging in humans^15,19,20^. For example, in our recent work in young adults with clinically normal audiograms (the current clinical gold standard for diagnosing sensorineural hearing loss from noise or other sources), we demonstrated that serum prestin levels have high test–retest reliability, using an experimental approach where serum was obtained at five time points over the span of multiple months^15^. We found that while serum prestin levels were stable over this time window on the individual level, there was considerable between-subject variation, despite all participants having clinically normal audiometric function. Moreover, our findings indicated a significant albeit weak relation to otoacoustic emissions (OAEs) (specifically, transiently evoked OAEs—TEOAEs), a clinical test used for ototoxic monitoring and newborn hearing screenings that is currently considered the most direct clinical measure of OHC function^21^. These earlier findings suggested that the broad range of circulating levels of prestin observed in this dataset could reflect individual differences in OHC function. However, no relations were found between serum prestin levels and audiometric thresholds, although both thresholds and TEOAE levels were in the clinically normal range. Because weak OAEs can sometimes be an indicator of small levels of OHC loss^22,23^, the relation between lower serum levels and weaker OAEs may suggest that serum prestin is sensitive to levels of OHC loss/damage too small to be detectable on the clinical audiogram.

The current paper follows up on these earlier findings by examining the relation between serum prestin levels and noise exposure levels in the same dataset of healthy young adults with normal audiograms. Noise exposure was measured objectively from body worn devices called noise dosimeters that the participant wore for three non-consecutive weeks to estimate how much noise they were exposed to on a daily basis. Our lab’s previous work using dosimeters has shown a range of exposures even in young adults with normal audiograms, from low levels of noise to daily exposure to hazardous sound levels^24–27^, with a similarly wide range emerging in this dataset.

**Figure 2:**
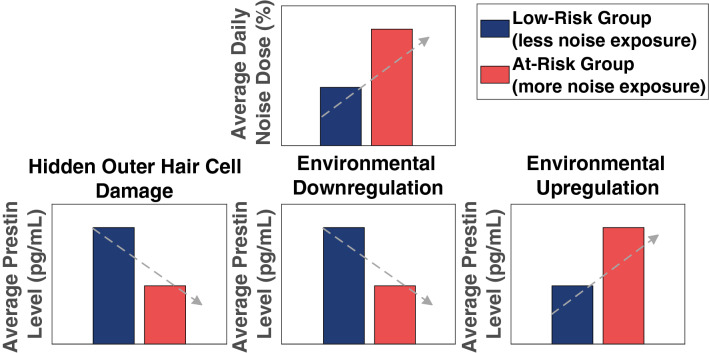
Three hypotheses for serum prestin and noise exposure. The predicted outcome of each hypothesis is illustrated by comparing groups with different levels of risk for noise-induced hearing loss, based on their average daily noise dose. In the current study, risk estimate was based on three weeks of objective environmental sound level measurements taken from body-worn noise dosimeters. We classified the young adults in our sample as at-risk if their daily noise doses exceeded 100% on average across the three weeks. Dose factors in both duration and intensity (in dBA) of exposure, with 100% dose defined as the upper limit of exposure that is considered safe from a regulatory standpoint. The gray arrows represent the direction of the predicted relationships between average daily noise dose and serum prestin levels, when noise exposure is treated as a continuous variable. The Hidden Outer Hair Cell Damage Hypothesis and Environmental Downregulation Hypothesis both predict a negative relation between serum prestin and noise levels, with individuals with higher noise doses having lower levels of serum prestin. However, the Hidden Outer Hair Cell Damage Hypothesis would also predict a negative relation between serum prestin and extended high frequency audiometry, while the Environmental Downregulation Hypothesis would not. Conversely, the Environmental Upregulation Hypothesis predicts a positive relation, with individuals with higher noise doses predicted to have higher serum prestin levels.

We propose three mechanistic hypotheses about how serum levels of prestin and noise exposure levels might relate (Fig. 2). If serum prestin levels are sensitive to subclinical OHC damage and this damage is due to routine noise exposure (see Refs.^28,29^ for studies examining subclinical cochlear damage in noise exposed populations), then serum prestin levels are expected to decrease with increasing levels of noise exposure. Hence, with this hypothesis, we would expect a negative relation between the two metrics, even in the absence of a clinically significant hearing loss as measured by an audiogram (“Hidden Outer Hair Cell Damage Hypothesis”). We refer to this type of OHC loss as “hidden” as it does not present itself as being outside clinical normative values for hearing thresholds on an audiogram or OAEs. If prestin levels are driven by subclinical OHC loss from noise exposure, then serum levels are expected to correlate with other metrics of subclinical hearing loss, such as extended high frequency (EHF) audiometry^30–32^.

An alternative hypothesis is that noise levels and serum prestin levels show a negative relationship, not from underlying OHC damage, but because of environmental pressures on the cochlear amplifier. Habitually loud environments may decrease the need for signal amplification, leading to the downregulation of prestin and lower protein turnover rate in healthy young adults who lead noisier lives (“Environmental Downregulation Hypothesis”). Alternatively, loud environments might be more metabolically stressful to the inner ear^33–35^, potentially driving higher protein turnover rate in young adults who regularly engage in loud activities and leading to higher noise levels positively correlating with higher serum levels of prestin (“Environmental Upregulation Hypothesis”). If serum prestin levels correlate with noise levels, but there is no corroboration of OHC damage from other measures of subclinical hearing loss, this would support the idea that serum prestin levels are modulated by environmental conditions in the healthy ear.

The current paper adopts an integrative approach to studying cochlear function that combines serum prestin levels, objective daily noise exposure levels, and clinical tests of OHC function to examine the relationship between circulating prestin levels and noise exposure in young adults with normal audiograms and TEOAEs, and evaluate the hypotheses described above. To obtain representative levels of noise, OAEs, and serum prestin levels for each participant, samples were taken multiple times per participant across a period of several months, and for all measures the average values were used in the analysis. In addition to performing a correlation analysis that treated noise levels continuously across the dataset, we also grouped participants based on their noise exposure levels, allowing us to compare the subgroup of participants most at risk for noise-induced hearing loss to a subgroup at lower risk.

## Results

### Prestin and noise exposure

**Figure 3:**
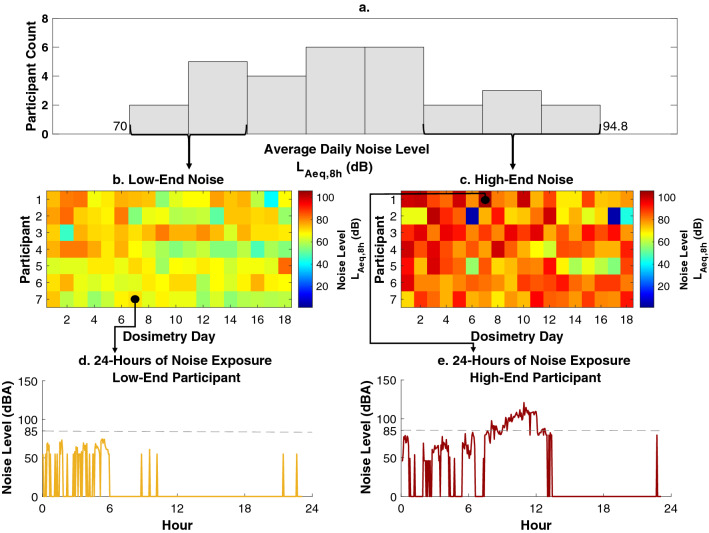
Noise exposure profiles. (**a**) A histogram of average daily noise levels across the dataset (70–94.80 L_Aeq, 8 h_ (dB)). Average daily noise levels (dB) were derived by averaging daily exposure across three weeks of measurement. For illustrative purposes, dB levels for individual measurement days are plotted for the participants in the lower and upper tiers of the histogram plot. Below this histogram, image plots show daily noise levels for the seven participants with the lowest noise levels (**b**) and the seven participants with the highest noise levels (**c**). For each participant, sound levels are shown for the 18-days for which 24-h long recordings were made (partial days are omitted from the image). To highlight the granularity of the data that went into calculating noise levels for each day, a sample 24-h time period is plotted for two participants, one with the lowest average daily noise dose (**d**) and one with the highest average daily noise dose (**e**). For consistency, each plot highlights noise levels measured on the Thursday during Round 2, the second measurement week, for both participants. The dotted gray line at 85 (dBA) represents the recommended exposure limit.

**Figure 4:**
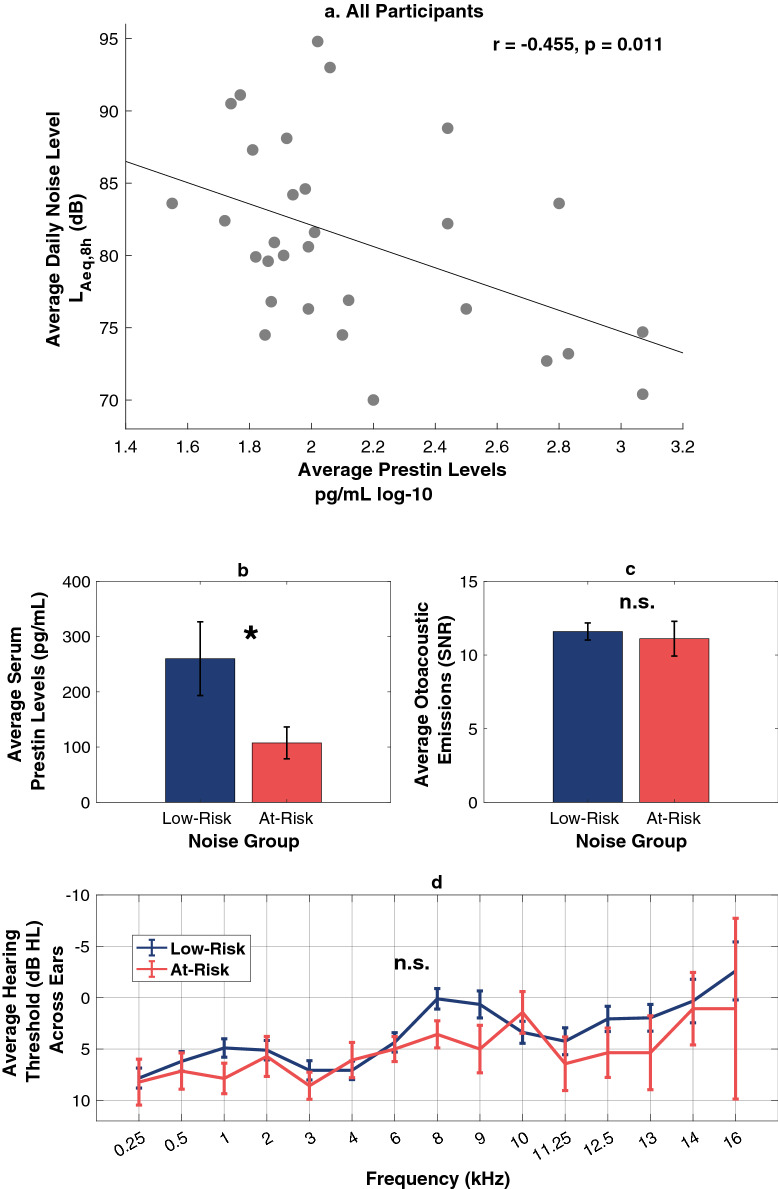
Serum prestin and noise levels across all participants, and separated by noise groups. (**a**) Average daily noise exposure level and average prestin levels show a negative relation, where individuals with higher levels of noise exposure have lower levels of serum prestin. This correlation aligns with the predictions of both the Hidden Outer Hair Cell Damage Hypothesis and the Environmental Downregulation Hypothesis. (**b**) Low-risk (< 100 dose %) and at-risk (> 100 dose %) noise groups differ significantly in average serum prestin levels, but not in average otoacoustic emissions signal-to-noise ratio (SNR) (**c**) or standard or extended high frequency (EHF) pure tone averages (PTA) (**d**). Error bars represent the standard error of the mean. *Significant at the p < 0.05 level; n.s. = not significant at the p < 0.05 level.

Average serum prestin levels ranged from 35.14 to 1186.23 pg/mL in our participants (n = 30) with a mean of 234.02 pg/mL. Average daily noise exposure levels ranged from 70.0 to 94.8 L_Aeq, 8 h_ (dB), with seven of the participants having average exposures at or above 85 dBA, placing them at greatest risk for hearing damage. The distribution of average daily noise levels (L_Aeq, 8 h_ (dB)), as well as day-by-day levels in our lowest- and highest-end participants, and hour-by-hour increments for single participants in the extreme of each group, are plotted in Fig. 3. See Table 1 for descriptive statistics. Our analysis shows a significant negative correlation between average serum prestin levels (pg/mL) and average daily noise exposure level (L_Aeq, 8 h_ (dB) (r = − 0.455, p = 0.011, Fig. 4A). That is, individuals with lower serum prestin levels tended to be more routinely exposed to higher levels of noise.

**Table 1:** Descriptive statistics.

	Average serum prestin levels (pg/mL)	Average daily exposure levels (L_Aeq, 8 h_ (dB))	Average daily noise exposure (%)
Mean	234.02	85.98	101.90
STD	309.63	6.67	197.23
SEM	56.53	1.22	1.38
ICC	0.98**	0.87**	0.73**
Minimum	35.14	70.00	82.39
Maximum	1186.23	94.80	111.48

*STD* standard deviation, *SEM* standard error of the mean, *ICC* intraclass correlations across 5 sessions (prestin) or 3 rounds (noise).

**p < 0.001.

### Noise exposure groups

As a complement to the correlation analysis, we compared groups of participants at the greatest risk of noise-induced hearing damage to those at lower risk based on three weeks of noise dosimetry, using a criterion from occupational settings to define: (1) an “At-Risk” group, who, by definition, was routinely exposed to noise in excess of the recommended daily exposure dose limit, with a daily average dose > = 100% (n = 7), and (2) a “Low-Risk” group who had daily average noise doses < 100% (n = 23) (Table 2). Daily dose is a metric that takes into account intensity (in dBA) of the sound and the amount of exposure time that allows us to compare exposure levels to safety standards. The noise dose is calculated such that a person exposed to 85 L_Aeq, 8 h_ (dB) will reach 100% of their daily recommended noise dose.

**Table 2:** Mean serum prestin levels, noise exposure, and audiometric measures, by group.

	Low-risk (n = 23)	At-risk (n = 7)
Male:female	8:15	2:5
Mean noise dose	26.84%	387.43%
Mean serum prestin levels	272.48 pg/mL	107.65 pg/mL
Mean standard PTA (across ears)	5.63 dB HL	7.14 dB HL
Mean extended high frequency PTA (across ears)	0.98 dB HL	3.48 dB HL
Mean otoacoustic emissions	10.94 SNR	10.33 SNR

*PTA* pure tone average.

This group comparison revealed that the At-Risk group had lower prestin levels, on average, than the Low-Risk group (Welsh’s t-test: t(27.095) = 2.133, p = 0.042) (Fig. 4B). These two groups, however, did not differ with respect to TEOAE signal-to-noise ratio (SNR, dB) (t(10.806) = 0.505, p = 0.624) (Fig. 4C) nor hearing thresholds in the standard audiometric range (using a pure tone average of thresholds at 0.5, 1, 2, and 4 kHz) (t(19.371) = − 1.560, p = 0.135). We also compared the groups with respect to their EHF audiometry (10–16 kHz), a measurement considered more sensitive to subclinical levels of OHC loss than the other two measures^31, 36^, and the groups did not differ on this measure either (a pure tone average of thresholds at 10, 12.5, 14, and 16 kHz) across ears (t(7.418) = − 0.551, p = 0.598). (Fig. 4D shows the full audiogram from 0.25 to 16 kHz.).

## Discussion

The goals of this study were to compare serum prestin protein levels and noise exposure levels in young-adult human subjects with a range of exposure levels but clinically normal hearing. Our findings show a significant negative relation between the two, where higher prestin levels paired with lower levels of routine noise exposure. This negative relationship is inconsistent with the Environmental Upregulation Hypothesis; we therefore focus the discussion on the Hidden Outer Hair Cell Damage Hypothesis and the Environmental Downregulation Hypotheses.

### Hidden Outer Hair Cell Damage Hypothesis

The results of our previous studies led us to hypothesize that lower levels of serum prestin could follow cochlear injury and be the result of fewer in-tact OHCs undergoing normal prestin production and turnover^15,17,18^. In our animal model studies, significant noise-induced loss of OHCs was confirmed histologically and manifested as noise induced changes in distortion product OAE levels and auditory brainstem response thresholds^17,18^. When serum prestin levels were measured six times over two weeks, a temporal pattern emerged in our experimental model that was characterized by a spike in circulating prestin levels just hours after high-level noise exposure, presumably in the aftermath of OHC death, followed by a gradual decline back to and eventually below baseline. This was interpreted as reflecting OHC loss leading to a smaller number of surviving OHCs continuing to undergo protein turnover^18^. Like in animal models, OHC damage is expected in humans exposed to dangerously high sound levels, and when this happens, serum levels are expected to be impacted. If sizeable, OHC loss is expected to correlate with elevated audiometric thresholds and reduced OAEs. However, if OHC damage is minimal, the loss may not be clinically detectable on an audiogram or using OAEs.

This brings us to the current study. To qualify for our study, participants were required to have normal bilateral audiometric thresholds (≤ 20 dB HL) in the standard frequency range (0.25–8 kHz) and have OAEs of sufficient integrity to pass a distortion product OAE screening protocol. This excluded participants with clinically-significant levels of OHC loss from being enrolled, but because standard hearing thresholds and OAEs can remain in the normal range in the early-stages of noise induced hearing loss (as seen in Ref.^37^), participants with small, subclinical levels of OHC damage would not have been excluded. Although OAEs are sensitive to clinically significant NIHL, previous work suggests they do not reliably predict the *exact* level of OHC loss, especially when losses are small^38,39^. Hence, the TEOAEs assayed in this study may not be sensitive enough to reveal changes in sensitivity, especially in revealing changes to high frequency hearing at the base of the cochlea. Once enrolled in the study, participants underwent detailed evaluation that included EHF audiometry (8–16 kHz) as a secondary indicator of early hearing damage^36^ and a three week dosimetry protocol to objectively measure daily average noise exposure levels and evaluate risk of noise-induced hearing loss. To capture sound levels representative of daily routines, participants wore a noise dosimeter clipped to their clothing for a total of ~ 21 days, spread across an academic year. In our sample of young adults, the distribution of daily average sound levels ranged from 70.0 to 94.8 L_Aeq, 8 h_ (dB). From the dosimetry measurements, we identified a subset of participants at the greatest risk for NIHL. By definition, the At-Risk group, average daily noise exposures exceeded 85 LAeq8h (dB) (100% dose), the recommended safety limit. A closer look at the data revealed that the At-Risk participants exceeded 85 L_Aeq, 8 h_ (dB) for at least five days and up to twelve days across the three-week measurement period, as the result of a variety of types of loud social and academic activities.

Consistent with serum prestin levels being a potential marker of early NIHL, we found that the At-Risk group had significantly lower serum prestin levels compared to the Low-Risk group. However, although the At-Risk group was regularly exposed to potentially hazardous sound levels, their TEOAEs levels and hearing threshold levels did not differ from the Low-Risk group, even when EHF audiometry was compared. Previous reports suggested that EHF audiometry is generally better than standard audiometry and OAEs at detecting the early stages, i.e., small levels, of OHC loss^31,36^. Moreover, although we previously found a weak correlation between serum prestin levels and TEOAEs^15^, unlike serum prestin, TEOAEs did not correlate with noise exposure levels in the current study. Thus, either noise-related OHC damage is too small to be detected by audiometry and TEOAEs but can be detected by serum prestin levels, or low serum prestin levels in the At-Risk group is being driven by a factor other than OHC loss, as we consider next.

### Environmental Downregulation Hypothesis

Across the auditory system, neurons adjust their responses based on the auditory environment, to reflect the mean, and other statistical characteristics, of environmental sound level patterns^40–43^. This fundamental, dynamic property of the auditory system emerges first in the auditory periphery^40^ where it likely mediated by cochlear processes^44^. The mechanisms of these adaptive inner ear processes, however, are not fully understood but may include pre- and post-synaptic rescaling of neurotransmitter release^44^, changes to cochlear amplifier gain^40^, and/or the cochlea up- or down-regulating its proteome to respond to changing environmental demands or internal conditions. In fact, pre-clinical models suggest that prestin expression is dynamically up- and down-regulated to compensate for cochlear damage and changes in acoustic input^45,46^.

Collectively, this led us to consider a second hypothesis, the Environmental Downregulation Hypothesis, for the relation between serum prestin and environmental noise exposure. The Environmental Downregulation Hypothesis posits that prestin expression is downregulated as part of the natural dynamics of OHCs responding to loud environmental conditions and the decreased need for cochlear amplification in loud environments. Accordingly, the At-Risk group may not have subclinical levels of OHC damage. What may instead differentiate them from the Low-Risk group is not the number of OHCs, but how much prestin is being expressed, and then turned over and released into circulation, by an approximately similar number of OHCs under different environmental sound level conditions. The significant correlation between serum prestin and environmental sound levels further suggests that prestin expression may scale in a graded fashion that reflects a range of environmental conditions, from quiet environments that are presumably “safe”, to loud environments that are potentially hazardous. Thus, noise induced OHC loss may not be necessary to explain patterns of serological dynamics if the “cochlear amplifier” adapts to environmental sound levels, i.e., prestin expression, a protein integral to cochlear amplification, scales accordingly.

One study that may support this adaptive regulation hypothesis involved the long-term administration of salicylate (aspirin), an ototoxic drug that can reversibly impede OHC electromotility. In this study^47^, cochlear function initially decreased after the initial dosage of salicylate. However, as long-term administration continued, prestin expression progressively increased, as did cochlear function. The authors suggested that in vivo prestin expression was dynamically upregulated to increase OHC electromotility during long-term salicylate administration. Further support comes from two studies^44,48^ that examined peripheral activity to sound levels below the traumatic sounds levels commonly used in animal research, including our own (e.g. Ref.^18^). In these studies, peripheral nerve activity was measured in an animal model following long-term continuous exposure to low-levels of noise (25–85 dB SPL) intended to approximate human noise pollution. Both studies showed a reduction in cochlear output with low-level noise exposure, measured via the amplitude of the compound action potential (CAP) of the auditory nerve; but importantly no changes to OAEs or OHC counts occurred. The average sound level needed for triggering this adaptive cochlear response was estimated to be 19 dB SPL^48^. This level is below the typical ambient levels in a home (e.g. Ref.^49^), suggesting that real-world acoustic experiences, even those that are relatively quiet and unlikely to damage the ear, could modulate cochlear dynamics. The authors also showed that CAP responses remained suppressed at least 12 h post exposure but returned to baseline levels 3–7 days after exposure occurred^48^. The authors interpreted these CAP findings as a “disruption” to cochlear function due to continuous sound exposure. Our results provide a potentially new interpretation by suggesting that environmental adaptations to cochlear function are not necessarily “disruptions” but could instead reflect healthy, normal cochlear dynamics, possibly linked to prestin expression. In this way, the OHCs may act like a “thermostat” that sets its protein production to match the average sound level of the environment, which in turn affects cochlear output to the central nervous system.

While both the Hidden Outer Hair Cell Damage and the Downregulation Hypotheses make the same negative prediction (lower prestin with high sound exposure), they deviate in what they would predict if environmental conditions changed. For example, if the At-Risk group adopted quieter lifestyles, or started wearing hearing protection, and their environmental sound levels changed to match the Low-Risk group, the Environmental Downregulation Hypothesis would predict that prestin levels would eventually increase for the At-Risk group to reach levels that match the Low-Risk group. These changes would likely not happen instantly^48^ or be instantaneously measurable in circulating blood. However, if no change occurred in serum prestin levels following a dramatic change to the acoustic environment, this would argue against the Environmental Regulation Hypotheses and favor the alternative that serum levels reflect OHC status. It is also important to acknowledge that both hypothesized mechanisms could operate in tandem in some cases: serum prestin could reflect OHC count and also the environmental pressures on the amplifier in an impaired system.

One limitation of our study is the small number of participants in the At-Risk group. This can be attributed to our recruitment approach, which focused on self-report of noise exposure in young adults with normal audiograms, rather than recruiting based on occupation or recreational activities. Overall, this led to an imbalance in the groups, though three weeks of dosimetry collection did help to confirm placement in “At- or Low-Risk” groups, and the correlation between noise levels and serum prestin levels suggests a continuous relationship. Further, while our three weeks of dosimetry showed strong/moderate intraclass correlations between weeks (see Table 1), these objective data cannot be generalized to noise histories from the past year or decade, and they do not factor in exposure from headphones during or before the study enrollment. Additionally, we were only able to test during hours that the phlebotomy clinic was open, hence, limiting testing to morning and weekdays may have influenced the sample demographic.

Another limitation that must be considered is whether the source of serum prestin is exclusively the inner ear. A recent study found that prestin is expressed in the heart, and that it may play a role in amplifying cardiac motor functions^50^. Our group also previously investigated the possibility of prestin being expressed in other tissues including heart, kidney, liver, brain, and skeletal muscle^51^. We found that expression in the heart was much lower than levels found in the blood stream. We interpreted that finding as consistent with the view that most of the prestin in circulation is likely from the inner ear. Of course, based on the data we present with the current study, it is not possible to rule out a cardiac contribution, but the relationship we found in our previous work between serum prestin levels and various auditory measures including auditory brainstem responses^17^, TEOAEs^15^ and word recognition scores^52^ suggests the inner ear as the main contributor. However, in our sample, environmental sound levels account for only 23% of the variance in the serum prestin levels. Hence, it is likely that serum samples reflect a mixture of inner-ear and cardiac contribution. We intend to pursue these questions in subsequent work.

In summary, in this study, participants who led louder lives were found to have lower levels of the protein prestin circulating in their blood. However, other measures of inner-ear function did not differentiate participants with the most and least noise exposure. This reveals the potential for serological evaluations of cochlear biomarkers to separate those at-risk for cochlear damage from those at less risk, and to provide new insights on inner ear dysfunction. These results encourage the continuing study of serum prestin levels, with the potential to improve early detection of hearing loss and yield greater insight into human cochlear dynamics. Larger-scale longitudinal approaches to studying serum prestin levels in at-risk populations are likely to further reveal the environmental and biological factors that collectively contribute to subclinical variations is OHC function.

## Materials and methods

### Experimental protocol overview

All experimental procedures were approved by the Institutional Research Board at the University of Connecticut, and participants provided their written informed consent prior to study enrollment. All experiments were performed in accordance with relevant guidelines and regulations. Testing occurred during the 2018–19 academic year, with each participant sitting for five test sessions (Sessions 1, 2, 3, 4, and 5, not including a first-day screening session) spanning over three separate, non-consecutive weeklong periods (Rounds 1, 2, and 3). Session 1 was conducted after a 14 + hour “quiet period”, where participants were asked to refrain from loud activities (e.g. music practice, large meetings, personal listening devices) to obtain audiometric thresholds unaffected by recent noise exposure or a temporary threshold shift. All testing occurred during an academic year while classes were underway. For each of the five sessions, blood samples were obtained.

### Participants

34 young adults (18–24 years old, mean = 20.26 years, 23 female), all undergraduate students at the University of Connecticut, initially enrolled in this repeated-measures study. Respondents to an advertisement were screened via a secured online questionnaire to confirm no history of chronic ear infections, ear surgery, a clinically-diagnosed hearing loss, hearing aid amplification or use, seizures or neuropathy, or past or current head trauma. All participants enrolled in the study were confirmed to have clinically-normal hearing bilaterally (air conduction audiometric thresholds ≤ 20 dB HL for octave frequencies from 0.25 to 8 kHz). All participants also passed a distortion product OAE screener (Madsen Alpha OAE screener, Otometrics, Inc.) and otoscopic exam. After confirming study eligibility, EHF audiometry was conducted via Sennheiser HDA200 circumaural headphones for test frequencies of 8, 10, 11.2, 12.5, 14, and 16 kHz. A tympanogram screener was also used to assess middle-ear function on all participants (Tympstar Middle Ear Analyzer, Grason-Stadler, Inc.), with no atypical responses noted. Additionally, participants completed an in-take survey that asked about current participation in a music ensemble and/or engagement in loud occupational or recreational activities.

### Blood draw procedures

Participants arrived at the lab in the morning each test day. Blood draws always occurred prior to the administration of any other hearing tests. Participants were escorted to and from the UConn Health Medical Services location in Downtown Storrs, where the venipuncture was performed by a certified phlebotomist who collected two 6.0 mL tubes of non-fasting blood samples (two red top tubes containing no anticoagulant or preservative) from the median cubital vein. Blood samples were left in their tubes, standing upright, for approximately 30 min at room temperature, before being transported back to our research facilities for further processing by a member of our research team who had undergone the necessary biosafety training. Blood samples were transferred from the red top tubes to microcentrifuge tubes before centrifuging. To separate the serum, the specimens were spun at 3000*g* for 10 min. After spinning, serum was collected via pipette and frozen at −80 °C until time of assay. At the conclusion of the study, samples were transported over dry ice from the UConn’s Storrs campus to the UConn Health campus in Farmington for final batch processing.

Prestin levels were measured in the serum using the MBS167508 ELISA kit (human prestin; MyBioSource, San Diego, CA) as described in the manufacturer’s instruction manual. A 1:5 dilution was prepared, and each serum sample was assayed in duplicate. The optical density in the wells of the ELISA micro-plate was measured at 450 nm using a Biotek ELx808 plate reader and data were compiled using the KCJunior software package (Bio-Tek Instruments, Inc., Winooski, VT). To avoid the risk for cross-plate variance, for each participant, their samples were processed in the same plate, with the technician blind to participant ID and all subject and test factors. The MBS167508 ELISA kit has a detection range of 10–3000 pg/mL with a sensitivity of 4.87 pg/mL. The manufacturer provides, in each kit, known standard concentrations of prestin, that are measured. The accuracy/reliability of each kit is tested individually against these standards. Deviations from these standards lead to rejection of the kit results, and only results from kits that passed this test are included in this study.

### Noise dosimetry protocol

To obtain a measure of routine noise exposure, we gathered three weeks of environmental sound level data from each participant. For each week, the protocol was similar to protocols established for prior studies in our lab^24–27^. Participants were trained to use their noise dosimeter and to record their daily activities into an activity log. Participants were instructed to clip their dosimeter onto their clothing near their ears and to leave the microphone inlet uncovered. When sleeping or showering, or during activities when the device might be damaged, participants were told they could remove the dosimeter but to keep it nearby.

The dosimeters were configured to an 85-dBA criterion level and 3-dB exchange rate, in conformance with the National Institute for Occupational Safety and Health (NIOSH) recommended criteria^58^, and a 70-dBA threshold. Dosimeters logged average sound level data in 3.75-min increments throughout the entire weeklong measurement period, yielding a maximum of 2688 samples logged over a full seven-day period. The calibration of all dosimeters was periodically checked to ensure that the instruments were operating properly. This was done by generating a continuous 1000-Hz narrowband signal at a nominal level of 90 dB SPL in an Audioscan Verifit test box and measuring its level with a type-1 sound level meter.

For each round, participants were scheduled to return in no less than one week (168 h) to turn in their dosimeter. At the end of each weeklong round of dosimetry, an experimenter collected the dosimeter and uploaded the sound level data to a lab computer using the ER200D Utility Suite software (Etymotic, version 4.04) in preparation for processing using an in-house MATLAB routine that separated the data by date. From each calendar day of dosimeter data, we derived two related measurements: an 8-h-normalized A-weighted equivalent continuous sound level L_Aeq, 8 h_ (in dB) and noise dose (expressed as a percentage) derived from this normalized sound level. L_Aeq, 8 h_ (dB) represents the total (A-weighted) sound energy within a specified time period in terms of an 8-h period. That is, for each day, we calculated the equivalent continuous sound level for an 8-h period that would contain the same sound energy as the full or partial day. Exposures at or exceeding the NIOSH recommended exposure limit of 85 L_Aeq, 8 h_ (dB), had noise doses >100%, and were considered potentially hazardous to hearing.

Participants repeated this weeklong protocol three times during non-consecutive weeks throughout the duration of the study. Three rounds of weeklong dosimetry, spread across the academic year, allowed for a representative measure of routine noise exposure^59^. To derive the average daily L_Aeq, 8 h_ (dB) noise level and average daily noise dose (%) across the three weeks, daily L_Aeq, 8 h_ and noise doses were logarithmically averaged across all days.

### Otoacoustic emissions protocol

TEOAEs were measured using HearID software (Mimosa Acoustics). A 50 dB SPL chirp served as the stimulus, band passed from 1 to 5 kHz, presented through an ER10C probe tip insert (Etymotic, Inc.) using a preset protocol (TE50_B2000_N60) within the HearID software that controlled the stimulus delivery, recording, and analysis process. TEOAE (SNR) was calculated automatically by subtracting the OAE noise level from the TEOAE magnitude.

### Statistical analyses

For all analyses, prestin levels (pg/mL), noise exposure levels (L_Aeq, 8 h_ (dB)), noise doses (%), and otoacoustic emissions (SNR) were averaged across sessions (the average prestin level of 5 blood draws, average OAE SNR of 5 test sessions, and logarithmic average noise levels and dose over three weeks). Audiometric thresholds (standard and EHF) used in the analyses were obtained after the baseline quiet period to avoid the impact of a temporary threshold shift. High intraclass correlations (two-way, absolute agreement) were found for both prestin levels and noise exposure, indicating high repeatability between sessions/rounds (see Table 1). Therefore, prestin levels were averaged across sessions, and dosimetry across weeks. Given that the prestin values range over several orders of magnitude, and to meet the assumption of normal residuals, their correlation data are plotted using a log scale, and subsequent correlation statistics are performed on log transformed values. Raw data (i.e. not log transformed) are used for prestin descriptive statistics and comparisons of means, and for noise level and dose in all analyses. TEOAE data and audiometric thresholds have also not been logged, having met conditions of normality.

Before conducting any analyses relating serum prestin and noise levels, an independent samples t-test examined whether prestin levels differed between the participant’s biological sexes. No difference was found (t(15) = 0.040, p = 0.970), and so males and females were pooled. We also confirmed that hearing thresholds were stable across time, calculating an intraclass correlation for pure tone averages across the five sessions (ICC = 0.893, p < 0.001). Out of a possible 34 participants with both serum samples and dosimeter data, two participants levels exceeded the ELISA kit range across all five sessions (> 3000 pg/mL). The large majority of our samples measured below 1000 pg/mL, similar to the values from the control group in another study examining serum prestin levels in humans^20^. Therefore, we treated these two high-end participants as outliers and dropped them from subsequent analyses, leaving us with n = 32. In addition, one participant left the study before its conclusion due to scheduling issues and another’s data was removed from analysis due to incomplete dosimeter data, resulting in a final n = 30.

A Pearson’s correlation was conducted between average prestin levels and average noise level across the full group of participants. Subsequent analyses were conducted between two groups of participants: at-risk and low-risk, where participants were at-risk if their daily noise doses exceeded 100% on average across the three weeks, the upper limit of exposure that is considered safe from a regulatory standpoint. Welsh’s t-tests were conducted between groups, due to unequal group sizes, for their prestin levels, OAEs, and standard and EHF audiometric thresholds. Statistical analyses were run with MATLAB version 9.5 (The MathWorks, Inc., Natick, MA).
